# Effect of imperceptible vibratory noise applied to wrist skin on fingertip touch evoked potentials – an EEG study

**DOI:** 10.14814/phy2.12624

**Published:** 2015-11-24

**Authors:** Na Jin Seo, Kishor Lakshminarayanan, Leonardo Bonilha, Abigail W Lauer, Brian D Schmit

**Affiliations:** 1 Division of Occupational Therapy, Department of Health Professions, Department of Health Sciences and Research, Medical University of South CarolinaCharleston, South Carolina; 2 Department of Mechanical Engineering, University of Wisconsin-MilwaukeeMilwaukee, Wisconsin; 3 Department of Neurology and Neurosurgery, Medical University of South CarolinaCharleston, South Carolina; 4 Department of Public Health Sciences, Medical University of South CarolinaCharleston, South Carolina; 5 Department of Biomedical Engineering, Marquette UniversityMilwaukee, Wisconsin

**Keywords:** EEG, finger, somatosensory evoked potential, stochastic resonance, tactile sensation, vibration

## Abstract

Random vibration applied to skin can change the sense of touch. Specifically, low amplitude white-noise vibration can improve fingertip touch perception. In fact, fingertip touch sensation can improve even when imperceptible random vibration is applied to other remote upper extremity areas such as wrist, dorsum of the hand, or forearm. As such, vibration can be used to manipulate sensory feedback and improve dexterity, particularly during neurological rehabilitation. Nonetheless, the neurological bases for remote vibration enhanced sensory feedback are yet poorly understood. This study examined how imperceptible random vibration applied to the wrist changes cortical activity for fingertip sensation. We measured somatosensory evoked potentials to assess peak-to-peak response to light touch of the index fingertip with applied wrist vibration versus without. We observed increased peak-to-peak somatosensory evoked potentials with wrist vibration, especially with increased amplitude of the later component for the somatosensory, motor, and premotor cortex with wrist vibration. These findings corroborate an enhanced cortical-level sensory response motivated by vibration. It is possible that the cortical modulation observed here is the result of the establishment of transient networks for improved perception.

## Introduction

The objective of this study was to investigate if cortical activity for sensing touch stimuli on the fingertip is affected by imperceptible white-noise vibration applied to wrist skin. Recent studies have demonstrated that fingertip tactile sensation changes with white-noise vibration applied to different locations in the upper extremity such as wrist, forearm, dorsum of the hand, or base of the palm (Enders et al. 2013; Hur et al. 2014; Lakshminarayanan et al. 2015; Wang et al. 2015). Continuous, imperceptible, white-noise vibration applied to wrist skin resulted in decreased tactile sensory threshold of fingertips, indicating improved fingertip touch sensation (Enders et al. 2013; Lakshminarayanan et al. 2015; Wang et al. 2015).

Sensation is important as a prerequisite for dexterous hand function including fine finger movements, gripping, and object manipulation (Johansson and Westling 1984; Augurelle et al. 2003; Monzee et al. 2003; Zatsiorsky and Latash 2004). Therefore, improved fingertip touch sensation with vibration has direct implications for a wearable sensory enhancer wristband to assist human performance in high-precision manual dexterity tasks as well as rehabilitation for those with a sensory deficit and impaired dexterity due to neurological problems (Seo et al. 2014).

Previous studies using imperceptible white-noise vibration have applied vibration directly to the fingertip to improve fingertip sensation (Liu et al. 2002; Kurita et al. 2013) or directly to the foot sole to improve foot sole sensation (Liu et al. 2002; Wells et al. 2005). However, the advantage of applying vibration to the wrist as opposed to the fingertips is that it exposes the entire finger/hand skin for relevant tactile stimuli during dexterous manual tasks and also does not interfere with object manipulation using fingers.

The neurobiological bases for this remote vibration enhanced sensory feedback are yet poorly understood. It is thought that this effect is mediated by the central nervous system, since imperceptible vibration applied to the wrist is unlikely to have reached the fingertip and increased the sensitivity of mechanoreceptors in the fingertip pad skin: Vibration loses more than 90% of its power as it travels 1–2 cm on the skin and approximately 99% of the power with a 6-cm travel due to the skin’s viscoelastic properties (Manfredi et al. 2012). While suprathreshold vibration may travel between the fingertip and wrist and activate remote mechanoreceptors (Delhaye et al. 2012; Libouton et al. 2012), the likelihood of activating remote mechanoreceptors becomes slim with subthreshold vibration, especially when the vibrating probe is surrounded by a ring, thus blocking the spread of vibration (Verrillo 1962) in the previous studies (Enders et al. 2013; Hur et al. 2014; Lakshminarayanan et al. 2015). In addition, manipulating the distance between fingertip and vibration location (e.g., fingertip–palm vs. fingertip–forearm) did not influence the results (Enders et al. 2013; Hur et al. 2014; Lakshminarayanan et al. 2015). Furthermore, increasing the vibration intensity to a suprathreshold level at remote locations only degraded fingertip tactile sensation (Lakshminarayanan et al. 2015), indicating that transmission of vibration from the wrist to fingertip could not have improved fingertip tactile sensation. Also, vibration is unlikely to directly lead to stimulation of the median nerve (responsible for fingertip sensation), since stimulation of skin areas innervated by the radial or ulnar nerve, not overlapping the median nerve, can lead to the same results (Enders et al. 2013; Hur et al. 2014; Lakshminarayanan et al. 2015).

In this study, we aimed to evaluate whether vibration enhanced tactile perception is mediated by cortical-level processing. We examined if imperceptible white-noise wrist vibration affects somatosensory evoked potential for fingertip touch. Specifically, we hypothesized that the peak-to-peak amplitude of the somatosensory evoked potential in response to suprathreshold fingertip touch would increase when imperceptible white-noise vibration is applied to the wrist.

## Methods

### Participants

We studied 20 self-reported right-handed healthy adults (10 males) with no neurological or psychiatric history, and no history of upper limb trauma. The mean age of the participants was 25 ± 5 years. The protocol was approved by the Institutional Review Board. Participants read and signed a written informed consent form before participating in the experiment.

### Procedure

The EEG somatosensory evoked potential in response to monofilament touch of the index fingertip was compared with versus without imperceptible white-noise vibration applied to the volar wrist.

#### Imperceptible wrist vibration

Imperceptible vibration was applied to the volar aspect of the left wrist using a vibrator, C-3 Tactor (Engineering Acoustics, Inc., Casselberry, FL). The vibrator was driven by white-noise signal low-pass filtered at 500 Hz, as described previously (Enders et al. 2013). The vibration intensity was adjusted to 60% of individual subjects’ sensory threshold at the wrist location determined at the beginning of the experiment. The sensory threshold is the minimum vibration intensity that a person can perceive and was determined using the method of ascending and descending limits (Ehrenstein and Ehrenstein 1999). All subjects reported that they could not feel the wrist vibration during the course of the EEG experiment.

#### Fingertip touch stimulation

The left index fingertip pad received touch stimulation by a monofilament delivered by a stepper motor triggered by a computer. The distance between the tip of the monofilament and the fingertip skin was adjusted so that the monofilament touches and bends slightly against the fingertip skin, in a similar manner compared with the clinical sensory assessment using the Semmes Weinstein monofilament test (Feng et al. 2009). The monofilament used here was similar to the 3.61 Semmes Weinstein monofilament, which represents a light touch with 0.2 g force that healthy adults should be able to perceive (Cooper and Canyock 2013). The reason that this study did not test a stimulus that becomes perceivable only with vibration is that somatosensory evoked potentials for perceived versus unperceived stimuli are known to be different (Auksztulewicz and Blankenburg 2013; Nierhaus et al. 2015) and the difference in the evoked potential would be attributable not only to vibration but also to perception (confounding). Thus, this study examined changes in the somatosensory evoked potential of a perceivable stimulus with vibration. The rationale is that vibration affects not only the tactile threshold, but also manual dexterity (Seo et al. 2014), suggesting changes in processing of perceived stimuli with vibration.

#### EEG acquisition

EEG signals were continuously recorded at 1 kHz using a 64-channel active electrode system (actiCAP, Brain Products GmbH, Gilching, Germany) and a Synamps^2^ amplifier system (Neuroscan, Charlotte, NC). The electrode position followed the international 10–20 system with an average reference and a ground at AFz. The EEG cap was placed on the subject’s head so that the Cz electrode was at the vertex. Each electrode site was hydrated using SuperVisc gel (Brain Products GmbH, Gilching, Germany). All electrodes’ impedance was below 20 kOhms. EEG signals were amplified, bandwidth filtered at 0.10–200 Hz, and recorded at 1 kHz using the Neuroscan software, Scan 4.5.

A total of 200 fingertip touch stimulations were presented with a random interstimulus interval of 4–5 sec through two continuous recordings of 9 min each. Each recording of 100 trials was composed of four blocks of 25 trials each. The imperceptible wrist vibration was on for two blocks, and off for the other two blocks. For vibration-on blocks, vibration was turned on 4–5 sec prior to the first touch stimulation and continued on throughout the block. Similarly, for vibration-off blocks, vibration was turned off 4–5 sec prior to the first touch stimulation and continued off throughout the block. The order of vibration blocks was randomized. Thus, each subject received 100 fingertip touch stimulations while wrist vibration was on and 100 fingertip touch stimulations while the wrist vibration was off. All subjects were able to perceive the monofilament touch of the fingertip. However, since the vibration was imperceptible, subjects did not know for which trials the wrist vibration was on.

**Figure 1:**
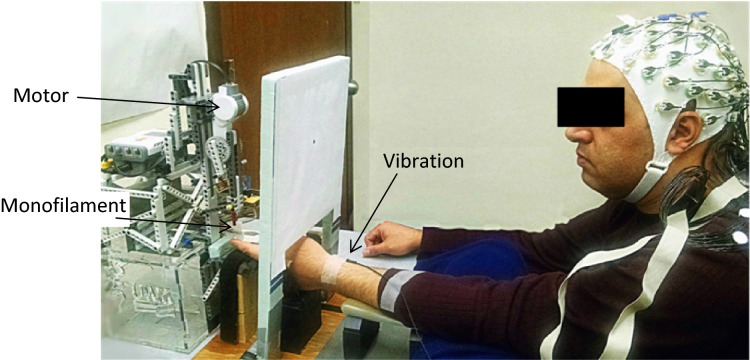
Experimental setup. The index fingertip pad received touch stimulation by a monofilament that was controlled by a motor connected to a computer, while EEG was recorded. Vibration to the wrist was turned on for the duration of the vibration-on trials or turned off for the vibration-off trials.

During EEG recording, subjects gazed at a fixation spot, wore ear plugs and a headphone to block sounds, and stayed relaxed (Fig.1). The motor moving the monofilament was contained in a foam structure to block the transmission of sound from the motor to the subject. All subjects reported that they could not hear the sound from the motor moving the monofilament. Subjects were seated with the left arm resting and left index fingernail fixed to stabilize the fingertip pad for the monofilament touch. The motor driving the monofilament and the finger receiving the touch were located behind a screen so that subjects could not see the monofilament’s movement relative to the fingertip.

#### EEG analysis

The EEG data were analyzed using MATLAB (The MathWorks, Natick, MA) and EEGLAB toolbox (Delorme and Makeig 2004). The data were band-pass filtered at 0.5–50 Hz to remove drifts and line noise. Independent component analysis was performed on the data to remove sources of artifacts using the ADJUST algorithm (Mognon et al. 2011). Data were then divided into epochs ranging from −100 to 600 msec relative to the stimulus onset (monofilament’s touch of the fingertip). The time period before the fingertip touch (−100 to 0 msec) served as the baseline brain activity. To remove additional artifacts, a moving window peak-to-peak threshold method in ERPLAB (Lopez-Calderon and Luck 2014) was used with a 200 msec moving window, a 100 msec window step, and a 100 *μ*V threshold, which resulted in rejection of an average 11% of trials (SD = 13%). The average somatosensory evoked potential was obtained by averaging remaining epochs for each subject for each condition.

The C4 electrode over the right somatosensory cortex contralateral to the stimulation site (Nierhaus et al. 2015) was of primary interest. Thus, while evoked potentials for all electrodes were visually examined, primary statistical analysis was performed for C4 electrode to compare mean peak-to-peak somatosensory evoked potential amplitudes between the vibration-on and vibration-off conditions in the subject group using a paired *t*-test. We tested the hypothesis that the evoked potentials for the vibration-on condition would be greater than the evoked potentials for vibration-off. Significance level of 0.05 was used. After obtaining a significant result for the mean peak-to-peak evoked potential amplitudes, the increase in the positive peak and decrease in the negative peak with vibration in the subject group were examined using paired *t*-tests with Bonferroni correction applied (with the significance level of 0.025).

**Figure 2:**
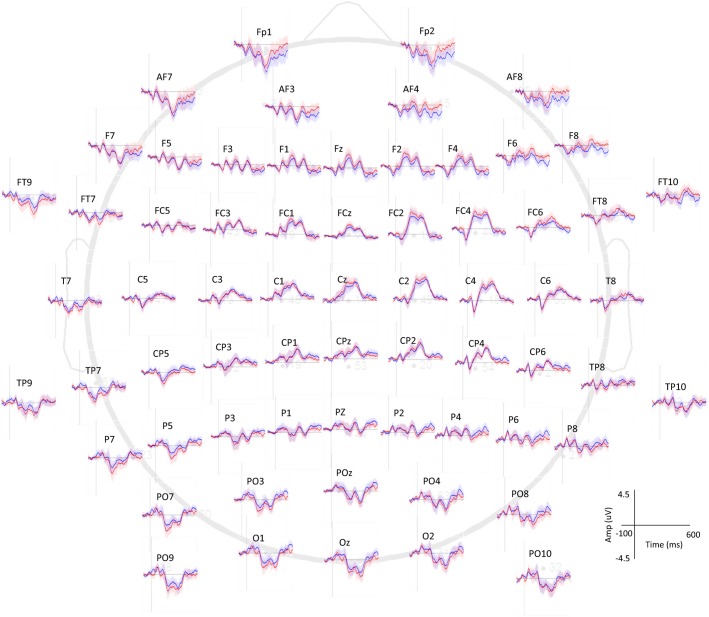
All electrodes’ average potentials after touch on the index fingertip pad (time = 0 msec) while imperceptible white-noise vibration was applied to the volar wrist (red) as compared to vibration turned off (blue). Mean potentials averaged for all subjects with 95% confidence intervals are shown.

As secondary analysis, the spread of the effect was examined for the C2, C4, C6, FC2, FC4, and FC6 electrodes representing the contralateral sensorimotor and premotor areas. Involvement of these areas in the later phase of the evoked potential was shown in previous sensory perception literature (Zhang and Ding 2010; Auksztulewicz and Blankenburg 2013) as well as from visual inspection of our results (Fig.2). Repeated measures ANOVA was performed to determine if the factors of electrode, vibration (on/off), and their interaction affected the positive peak of the evoked potential.

In addition, source reconstruction was performed to evaluate the anatomical location of the evoked potential generators. The whole brain’s cortical current sources were modeled using Brainstorm (Tadel et al. 2011) on a standard 3D brain model (Colin27: MNI brain with 1 mm^3^ isotropic voxel size) for the somatosensory evoked potential epoch period (−100 to 600 msec) for each subject and condition. Source reconstruction was performed on the evoked EEG data encompassing all channels (with the same filter settings 0.10 to 200 Hz) with 1 msec time bin. Forward modeling was conducted using OpenMEEG, which uses the symmetric boundary element method (Gramfort et al. 2010), and inverse modeling of the sources was constructed using a whitened and depth-weighted linear L2-minimum norm estimates (wMNE) algorithm (Tadel et al. 2011). The whole brain EEG sources were then obtained for the signal comprised within the 10 msec time bin around the negative and positive C4 evoked potential peaks (5 msec before and after the peak). The voxel-wise sources in standard MNI space were exported to nifty format, spatially smoothed with an isotropic three-dimensional Gaussian Kernel of 10 mm, and averaged across subjects for each condition to visually compare between the two vibration conditions.

## Results

**Figure 3:**
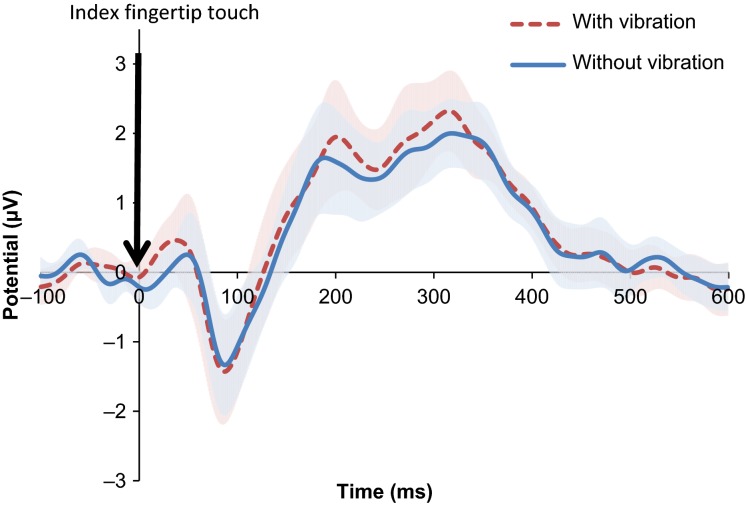
Somatosensory evoked potential after touch on the index fingertip pad while imperceptible white-noise vibration was applied to the volar wrist (red segmented line) as compared to vibration turned off (blue solid line). Mean potentials with an upper or lower bound 95% confidence interval at C4 electrode averaged for all subjects are shown.

**Figure 4:**
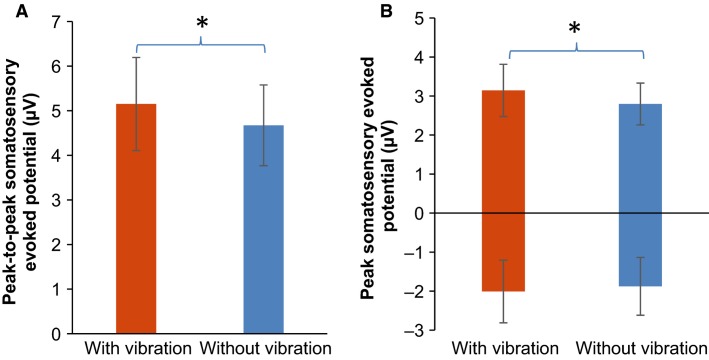
Mean peak-to-peak somatosensory evoked potential at C4 electrode after touch on the index fingertip pad while imperceptible white-noise vibration was applied to the volar wrist as compared to vibration turned off. Mean of 20 subjects’ mean peak-to-peak somatosensory evoked potentials are shown with 95% confidence intervals (A). In addition, the mean positive and negative peaks for 20 subjects were compared separately (B). The asterisk indicates a statistically significant difference between the two vibration conditions.

**Figure 5:**
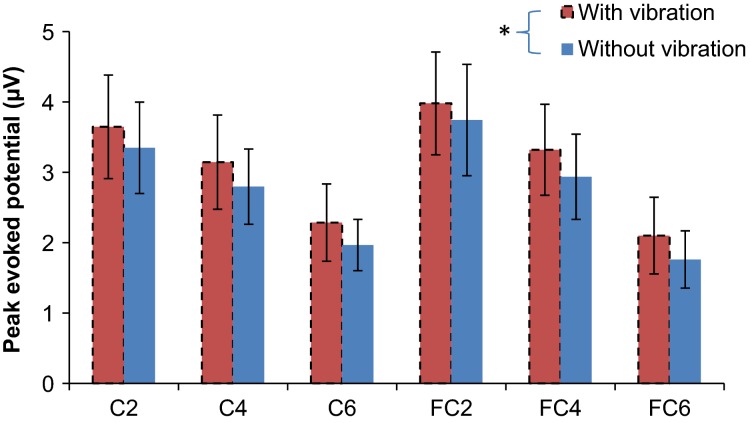
Peak somatosensory evoked potentials at C2, C4, C6, FC2, FC4, and FC6 electrodes after touch on the index fingertip pad while imperceptible white-noise vibration was applied to the volar wrist as compared to vibration turned off. Mean of 20 subjects’ mean positive peak of the somatosensory evoked potentials are shown with 95% confidence intervals.

Potentials after index fingertip touch with versus without wrist vibration are shown for all electrodes in Figure2. Specifically, evoked potentials for the C4 electrode averaged for all subjects are shown for the vibration-on and -off conditions (Fig.3). Peak-to-peak amplitudes of the somatosensory evoked potential after touch of the index fingertip pad averaged for all subjects are compared between the two vibration conditions in Figure4A. The peak-to-peak evoked potential was significantly greater while the imperceptible white-noise vibration was applied to the volar wrist compared to while the vibration was turned off (*P *=* *0.003, Fig.4A). The initial negative peak was not significantly larger with the vibration than without (*P *=* *0.180), whereas the late positive peak was significantly larger with the vibration than without (*P *=* *0.024, Fig.4B). The negative peak occurred at 85 ± 10 msec (95% confidence interval) and 93 ± 16 msec for the vibration-on and -off conditions, respectively (*P *=* *0.113), and the positive peak occurred at 277 ± 31 msec and 274 ± 31 msec for the vibration-on and -off conditions, respectively (*P *=* *0.376). The secondary analysis showed that the vibration significantly affected the positive peak of the evoked potential for all six electrodes encompassing the sensorimotor and premotor areas (Fig.5, *P* = 0.004 for the vibration effect and *P* = 0.999 for the vibration and electrode interaction).

**Figure 6:**
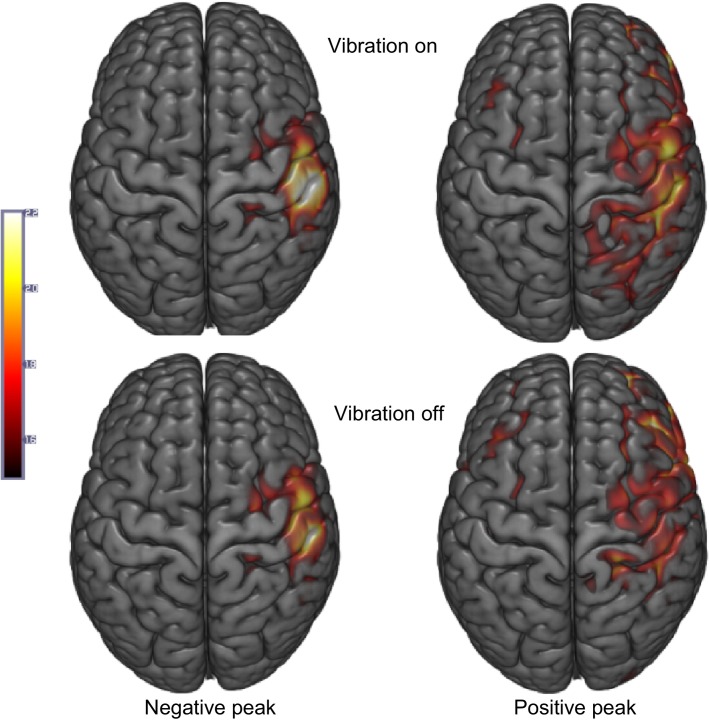
Source localization for the vibration-on (top) and vibration-off (bottom, control) conditions. Subject-averaged brain activity for the early negative peak (left) and the late positive peak (right) compared to the baseline (100–0 msec before touch) is shown for both vibration conditions.

Source localization indicates activity on the sensorimotor area after fingertip touch (Fig.6). Specifically, changes in brain activity at the early negative peak and late positive peak of the C4 electrode somatosensory evoked potential after touch of the fingertip pad compared to the baseline (average across 100 to 0 msec before touch), averaged for all subjects, are shown for the vibration-off and -on conditions. A greater sensorimotor neural recruitment is observed in the vibration-on condition, especially during the late positive evoked potentials (Fig.6 right).

## Discussion

The result of this study provides evidence that imperceptible white-noise vibration applied to the volar aspect of the wrist affects cortical processing of fingertip tactile stimuli. Specifically, peak-to-peak somatosensory evoked potentials at the somatosensory cortex increased with wrist vibration. This increased peak-to-peak amplitude was due to increase in the positive peak in the later phase (after 200 msec), not the negative peak in the earlier phase (∼100 msec) of the cortical sensory processing. This increased later phase positive peak was spread across the somatosensory, motor, and premotor cortex. Change in conscious attention could not have been involved because subjects did not feel the vibration throughout the EEG recordings and the order of vibration-off and -on blocks were randomized.

This observation supports the modulation of cortical-level somatosensory processing during manipulation of vibratory feedback, providing the neurobiological basis for its use in rehabilitation. These findings challenge the typical assumption that imperceptible vibration at wrist, for instance from resting the hand on a table, has no influence on finger sensation. They also support the previous findings of remote vibration-induced changes in fingertip tactile perceptual sensory threshold (Enders et al. 2013; Wang et al. 2015) and associated motor behavior (Hur et al. 2014; Seo et al. 2014), supporting further investigation for use of wrist vibration to affect finger sensation for various applications.

The significant increase in the later component, but not in the earlier component of the somatosensory evoked potential (Fig.4B) indicates that vibration affects conscious experience of the stimuli. The early component of the somatosensory evoked potential originates from the arrival of the thalamo-cortical volley (Allison et al. 1991; Nierhaus et al. 2015) and is representative of stimulation strength which in this study was constant between the vibration-on and -off conditions. While perithreshold stimuli can evoke varying amplitudes of the early component potentially due to variability in neuronal firing and the amplitudes are associated with awareness (Auksztulewicz and Blankenburg 2013), the present study used a suprathreshold stimulus that may be less affected by variability in neuronal firing. Similarities in the negative evoked potential at this time point suggest that the evoked signal reaching cortical levels is similar with or without vibration. On the other hand, the later components correlate with conscious experience and recurrent processing within the network of somatosensory and premotor cortices (Zhang and Ding 2010; Auksztulewicz and Blankenburg 2013). The wrist vibration appears to have affected this conscious experience and recurrent processing of the finger tactile stimulus. With vibration, increased responses in the contralateral C and FC electrodes associated with the late component of the evoked potential support the idea that vibration has an effect on premotor areas of the cortex.

It is possible that the cortical modulation observed here is the result of the establishment of transient networks for recurrent processing and improved perception. Sensory noise has been shown to increase phase synchronization within and between EEG cortical sources (Kitajo et al. 2007; Lugo et al. 2008; Ward et al. 2010), suggestive of establishment of networks (Ward 2009; Ward et al. 2010) for somatosensory processing. Such phase synchronization among brain areas is associated with improved sensory perception: Visual or auditory noise in one eye or one ear improves detection with the other eye or the other ear (Kitajo et al. 2007; Lugo et al. 2008; Ward et al. 2010). Even enhanced finger tactile sensory threshold was reported with auditory noise (Lugo et al. 2008). Thus, the wrist vibration could have affected phase synchronization related to somatosensory processing of the finger stimuli.

In contrast to this body of literature describing the effect of background sensory noise on detection of other sensory signal, brief imperceptible sensory stimulation alone (without other sensory signal to detect) has been shown to transiently reduce BOLD signals suggesting focal deactivation or inhibition (Blankenburg et al. 2003), reduce functional connectivity between the primary somatosensory area (SI) and frontoparietal areas and increase EEG alpha frequency power for the somatosensory area (Nierhaus et al. 2015) indicative of “cortical idling” (Pfurtscheller et al. 1996), resulting in impediment in sensory processing for the finger area receiving the imperceptible electrical stimulation (Blankenburg et al. 2003). The finding of the present study may not be in direct contradiction with these previous studies, as the imperceptible vibratory stimulation of the wrist could have induced a focal deactivation of the wrist area in the somatosensory cortex and spared neural resources for better sensing of other hand areas such as fingers, as in temporary deafferentation (Weiss et al. 2004, 2011; Sens et al. 2012). In previous deafferentation studies, numbing of forearm skin resulted in improved fingertip sensation assessed by the Grating orienting task and improved hand dexterity assessed by the Shape-sorter-drum task (Weiss et al. 2011; Sens et al. 2012) as well as increased evoked magnetic field for fingertip tactile stimulation and expansion of cortical representations for the fingers (Sens et al. 2012).

Taken together, our findings complement previous observations by corroborating that changes in sensory processing due to interfering stimuli occur as a result of modulation of cortical-level networks. The recruitment of neural resources may depend on the underlying neural circuitry and anatomical distributions of cortical representations. Disturbance affecting adjacent but overlapping cortical areas may lead to destructive interference. For example, across areas related to the index and middle fingers with a cortical overlap (Krause et al. 2001), impaired sensing for the index finger, either by constant frequency tactile stimulation (Ragert et al. 2008) or imperceptible electrical stimulation (Taskin et al. 2008), resulted in impaired sensing for the middle finger (Ragert et al. 2008) and decreased BOLD signal in response to middle fingertip touch (Taskin et al. 2008). Conversely, when cortical areas are adjacent but separated such as between wrist and fingertip, it is possible that one area’s deactivation leads to adjacent areas’ increased activity (Weiss et al. 2004). However, it is also postulated that when the cortical areas are far away from each other (e.g., fingertip and upper arm or leg), the effect would not sustain.

In conclusion, the findings from this study indicate that enhanced sensory response motivated by vibratory sensory noise is related to cortical modulation, possibly as a result of the establishment of transient networks for improved perception. This mechanism could be explored for further use in neural rehabilitation. For instance, patients with impaired sensorimotor function who still have the two somatotopic areas adjacent to each other with residual tracts may use this sensory vibration to enhance their sensory experience and subsequent motor control. This study examined rather immediate effects of vibration, not the effects of long-term exposure to vibration. With long-term exposure of hours and days as in rehabilitation settings, dynamic changes may occur with sensitization or adaptation, which needs to be addressed before use of vibration in a long-term rehabilitation setting.
